# Supplementary material for: Iturin A Rescued STb-R-Induced Pork Skeletal Muscle Growth Restriction through the Hypothalamic-Pituitary-mTORC1 Growth Axis
Source: Animals (Basel). 2022 Jun 17;12(12):1568. doi: 10.3390/ani12121568 (PMC9219473; doi:10.3390/ani12121568)
Supplement: Supplementary file 1 [file animals-12-01568-s001.zip › animals-1669794-supplementary.pdf]

Table S1. Ingredient composition and nutritional levels of the diets (air-dry basis, %)

Ingredients	Content	Nutritional levels	Content
Corn	33.66	Digestible energy, MJ/kg	14.23
Soybean meal	24.00	Crude protein[2]	19.15
Extruded corn	20.00	Lysine	1.30
Flour	10.00	Methionine + cystine	0.58
Fermented soybean meal	4.00	Threonine	0.71
Soybean oil	2.00	Tryptophan	0.23
Dicalcium phosphate	0.80	Calcium	0.53
Limestone power	0.72	Phosphorus	0.47
Mildew preventive	0.05		
Sodium chloride	0.40		
L-Lysine hydrochloride	0.37		
Premix[1]	4.00		
Total	100.00		

[1] The premix provided the following nutrients per kilogram of diet: Fe 101.00 mg; Cu 15.00 mg; Mn 42.00 mg; Zn 102.00 mg; I 0.60 mg; Se 0.30 mg; VA 10500.00 IU; VD_3 2100.00 IU; VK_3 3.50 mg; VB_1 2.24 mg; VB_2 9.80 mg; VB_6 2.80 mg; VB_{12} 0.05 mg; Folic acid 1.04 mg; Niacin 35.00 mg.

[2] Crude protein was analyzed. Other values were calculated.

Table S2: Primers used for qRT-PCR

Gene	Primer	Sequence (5′- 3′)	Size (bp)
GHRH (XM_005672915.3)	sense	CTGCTCTGGGTGTTCTTCCT	262
	antisense	CCATCTGCTTTTGGTCTGC	
GH (NM_213869.1)	sense	GTTTGGCACCTCAGACCG	279
	antisense	CCCAGCAACTAGAAGGCACAGC	
GHRHR (XM_021078507.1)	sense	CCCCTGCCCGGCTTTCTTCT	558
	antisense	CCCACCCACGTGCCAGTGAA	
GHR (XM_021076575.1)	sense	TGAGCCCATTTGCATGTGAAG	322
	antisense	TCTGAGCCTTCAGTCTTTTCATC	
IGF-1 (XM_005664199.3)	sense	GGACCCGAGACCCTCTGCGGG	210
	antisense	GGCCGACTTGGCGGGCTTG	
IGF-1R (XM_021082920.1)	sense	TACGTCCAGGCCAAAACGA	287
	antisense	GCGACGCCTTCATAAACCA	
GAPDH (NM_001206359.1)	sense	GGTCGGAGTGAACGGATTT	246
	antisense	CATTTGATGTTGGCGGGAT	
β-actin (XM_003124280.5)	sense	CTGGAACGGTGAAGGTGACA	165
	antisense	AAGGGACTTCCTGTAACAATGCA	

GHRH = growth-hormone-releasing hormone; *GH* = growth hormone; *GHRHR* = growth-hormone-releasing hormone receptor; *GHR* = growth hormone receptor; *IGF-1* = insulin-like growth factor 1; *IGF-1R* = insulin-like growth factor 1 receptor; *GAPDH* = glyceraldehyde-3-phosphate dehydrogenase.

Table S3. The effect of Iturin A and STb-R on the porcine hindquarter muscles (g).

Item	Control	Iturin A	STb-R	Iturin A+STb-R	*P*-value
Middle gluteus medius muscle	24.65±1.69	27.57±2.12	25.70±2.00	26.25±1.04	0.505
Medial vastus muscle	45.95±2.59	48.58±2.30	44.68±3.63	47.97±1.34	0.100
Biceps femoris muscle	27.2±2.21	29.52±1.46	23.63±2.91	28.57±1.65	0.488
Tensor fascia lata muscle	23.9±0.72	25.44±1.38	25.66±1.55	27.07±2.35	0.182
Cranial tibial muscle	71.05±4.26	71.85±2.00	67.38±2.73	75.33±2.33	0.305
Peroneus tertius muscle	18.95±1.29	19.25±0.80	19.1±1.01	20.96±1.17	0.193
Gracilis muscle	14.55±0.87	16.07±0.51	14.35±1.35	15.73±0.54	0.252
Adductor muscle	14.86±0.77	15.75±0.35	14.4±0.63	14.93±0.68	0.376
Long peroneal muscle	11.85±1.17	13.4±1.24	10.4±1.10	13.1±1.04	0.209

Data were presented as mean ± SEM (n=7).

Table S4. The effect of Iturin A and STb-R on the porcine forequarter muscles (g).

Item	Control	Iturin A	STb-R	Iturin A +STb-R	*P*-value
Infraspinatus muscle	85.58±4.30	88.05±3.31	77.6±7.53	87.1±4.42	0.302
Cranial portion of infraspinatus muscle	145.64±12.83	134.94±3.35	157.07±13.06	164.79±12.62	0.127
Supraspinatus muscle	24.3±1.45	24.88±2.35	24.73±2.48	22.03±1.21	0.389
Subclavius muscle	4.08±0.31	4.04±0.15	3.82±0.57	4.21±0.26	0.709
Long head of triceps of brachii muscle	8.96±0.92	9.6±0.34	9.31±0.27	9.99±0.48	0.278
Lateral head of triceps of brachii muscle	30.17±0.94	28.68±1.33	28.75±1.33	28.75±1.77	0.251
Extensor carp iradialis muscle	4.36±0.57	4.27±0.15	4.16±0.39	4.95±0.14	0.264
Latissimus dorsi muscle	94.4±7.09	98.7±6.41	96.78±3.47	90.53±4.50	0.614

Data were presented as mean ± SEM (n=7).
